# Designing the CORONIS trial. Why a non-regular fractional factorial design?

**DOI:** 10.1186/1745-6215-14-S1-O57

**Published:** 2013-11-29

**Authors:** Pollyanna Hardy, Ed Juszczak, Barbara Farrell

**Affiliations:** 1 National Perinatal Epidemiology Unit, University of Oxford, Oxford, UK, on behalf on the CORONIS Trial Collaborative Group, Oxford, UK

## Introduction

Caesarean section is one of the most commonly performed operations worldwide. A variety of surgical techniques for all elements of the operation are used. Many of them have not been rigorously evaluated in randomised controlled trials. The CORONIS Trial set out to simultaneously examine five elements of the caesarean section operation in seven low- to middle-income countries, using a novel adaptation of the factorial design.

## Design

During the planning stages of CORONIS, five pairs of caesarean section techniques were agreed upon using a consensus process, for evaluation in a factorial design. It subsequently became apparent that, for pragmatic reasons, not all five pairs could be randomly allocated in all participating countries. Therefore, each participating site was assigned 3 of the 5 intervention pairs. In addition it was agreed that examining interactions was not important. This led to the adoption of a non-regular fractional factorial design, the first known use of its kind in a clinical trial setting. This presentation will explain how pragmatic considerations influenced the design of the CORONIS Trial and their implications on the sample size, randomisation, central monitoring, conduct and analysis.

## Conclusions and recommendations

Our experience of using a complex trial design to deliver robust and reliable results was successful, but required a team of innovative researchers and clinicians. We would recommend undertaking such a design only with extreme caution.
